# Screening and evaluation of key genes in contributing to pathogenesis of hepatic fibrosis based on microarray data

**DOI:** 10.1186/s40001-020-00443-0

**Published:** 2020-09-17

**Authors:** Furong Wu, Lijuan Ning, Ran Zhou, Aizong Shen

**Affiliations:** Department of Pharmacy, The First Affiliated Hospital of USTC, Division of Life Sciences and Medicine, University of Science and Technology of China, Hefei, 230001 Anhui People’s Republic of China

**Keywords:** Hepatic fibrosis, Key genes, Functional enrichment analysis, Protein–protein interaction

## Abstract

**Background:**

Hepatic fibrosis (HF), which is characterized by the excessive accumulation of extracellular matrix (ECM) in the liver, usually progresses to liver cirrhosis and then death. To screen differentially expressed (DE) long non-coding RNAs (lncRNAs) and mRNAs, explore their potential functions to elucidate the underlying mechanisms of HF.

**Methods:**

The microarray of GSE80601 was downloaded from the Gene Expression Omnibus database, which is based on the GPL1355 platform. Screening for the differentially expressed LncRNAs and mRNAs was conducted between the control and model groups. Then, Gene Ontology (GO) and Kyoto Encyclopedia of Genes and Genomes (KEGG) analyses were performed to analyze the biological functions and pathways of the DE mRNAs. Additionally, the protein–protein interaction (PPI) network was delineated. In addition, utilizing the Weighted Gene Co-expression Network Analysis (WGCNA) package and Cytoscape software, we constructed lncRNA-mRNA weighted co-expression networks.

**Results:**

A total of 254 significantly differentially expressed lncRNAs and 472 mRNAs were identified. GO and KEGG analyses revealed that DE mRNAs regulated HF by participating in the GO terms of metabolic process, inflammatory response, response to wounding and oxidation–reduction. DE mRNAs were also significantly enriched in the pathways of ECM-receptor interaction, PI3K-Akt signaling pathway, focal adhesion (FA), retinol metabolism and metabolic pathways. Moreover, 24 lncRNAs associated with 40 differentially expressed genes were observed in the modules of lncRNA-mRNA weighted co-expression network.

**Conclusions:**

This study revealed crucial information on the molecular mechanisms of HF and laid a foundation for subsequent genes validation and functional studies, which could contribute to the development of novel diagnostic markers and provide new therapeutic targets for the clinical treatment of HF.

## Background

Hepatic fibrosis (HF), a common health issues worldwide, usually progresses to liver cirrhosis, primary liver cancer and then results in death [1–3]. Despite the impact of HF, it is regretful about note that an optimal treatment has yet to be identified. The good news is that advances in molecular biotechnology and high-throughput technology have positioned us on the frontier of understanding the possible molecular mechanism of HF at the gene and protein levels [4–6]. However, a comprehensive understanding of the pathogenesis underlying HF still remains to be elucidated, due to its complexity, especially the complicated regulatory mechanisms of gene expression. Hence, understanding the underlying pathophysiology of HF, and the screening of molecular markers in the development of therapeutic targets remains critical.

More and more evidence has indicated that, rather than being transcriptional noise, lots of non-coding RNAs (ncRNAs) can affect the expression levels of target genes [7, 8]. Long non-coding RNAs (lncRNAs), one of a ncRNAs, which play critical roles in transcription, splicing and translation [9]. However, up to now, compared with mRNAs, the function of lncRNAs has not been well annotated [10, 11]. Therefore, to research the roles of lncRNAs by studying the related to targeting genes such as mRNAs, whose functions have been known, have been certificated an efficient way in many kinds of diseases [12–14].

In the current study, we used microarray data to identify novel HF-related lncRNAs and mRNAs. Furthermore, bioinformatics technologies, such as Gene Ontology (GO), Kyoto Encyclopedia of Genes and Genomes (KEGG) analyses, protein–protein interaction (PPI) network and weighted gene co-expression network analysis (WGCNA), were applied to analyze the differentially expressed lncRNAs (DELs) and mRNAs (DEMs). The findings may provide in-depth molecular insight into the pathophysiology of HF.

## Materials and methods

### Raw data

The gene expression data of GSE80601 was downloaded from the National Center for Biotechnology Information (NCBI) Gene Expression Omnibus (GEO, http://www.ncbi.nlm.nih.gov/geo/) database, which is based on the Affymetrix Gene Chip Mouse Exon 1.0 ST Array. It has a total of 10 data in the dataset, including 5 samples in the control group and 5 samples in the HF group, which were induced by carbon tetrachloride [15].

### Gene chip probe re-annotation

A number of lncRNAs represented on the Affymetrix microarray were identified based on the lncRNAs classification pipeline constructed in previous research [16]. First, we gained the latest version of the NetAffx Annotation File (MoEx-1_0-st-v1 Probeset Annotations, CSV Format, Release 36 93 MB, 7/6/16) from the Affymetrix official website. The annotation file was mapped to the MoEx-1_0-st-v1 probe sets ID. Second, among the probe sets from the Refseq database, the IDs beginning with ‘NP’ were wiped off, while the transcript IDs labeled with ‘NR’ were retained. For the probe sets from the Ensembl database, Affymetrix microarray IDs and the corresponding gene type were converted to Ensembl IDs by using the online software BioMart. The probe sets from NONCODE were reserved. Only genes that were annotated as ‘lincRNA’, ‘sense_intronic’, ‘processed_transcript’, ‘antisense’, ‘sense overlapping’, ‘3prime_overlapping_ncRNA’, and ‘misc_RNA’ were retained. Next, probe set IDs annotated as ‘microRNA’, ‘snoRNAs’, ‘pseudogenes’ and other small RNAs were removed.

### Data preprocessing

Based on the annotation of GPL1355 platform, we converted the expression data of probes to the corresponding gene symbols. The average expression value, used for genes corresponding to multi-probes, was calculated using Aggregate function of R, the missing values of probes were added via the KNN method of Impute package of R [17]. Next, using the preprocessCore package of R, quantile normalization was carried out [18]. We used the above steps to get the expression matrix.

### Identification of DELs and DEMs

After the raw data from the mRNA-lncRNA Affymetrix microarray were selected, we screened the differential probes by applying the Limma (linear models for microarray data) package in R via *T*-test with the screening criteria: fold Change (FC) > 1.5 and *P* value < 0.05. Then, the differential probes representing the DELs and DEMs were re-annotated. Based on these two steps, the DELs and DEMs were obtained.

### GO and pathway enrichment analysis

GO biological processes term and KEGG pathway for DEMs were performed using Cytoscape-bingo and Database for Annotation, Visualization and Integration Discovery (DAVID, https://david.ncifcrf.gov/), respectively. Then, A FDR was calculated to correct the p-value and FDR < 0.05 was selected as the threshold.

### Construction of PPI network

The online String database (Search Tool for the Retrieval of Interacting Genes, http://string-db.org/) was used to analyze the interactions of proteins. Genes without connection to other genes were removed, with a cut-off criterion of the combined score > 0.90. Then, the PPI network was delineated by Cytoscape (http://cytoscapeweb.cytoscape.org/).

### Construction of lncRNA-mRNA WGCNA

The R package WGCNA was applied for network constructions [19]. The steps of network construction were as follows: (1) Network construction: the lncRNA/mRNA weighted co-expression network is fully specified by its adjacency matrix amn, where amn encodes the network connection strength between nodes m and n. To calculate the adjacency matrix, the default approach defines the co-expression similarity Smn as the absolute value of the correlation coefficient between nodes of m and n: Smn = |cor am, an)|. The weighted adjacency amn between two genes is proportional to their similarity on a logarithmic scale with b^3^0.8, log (amn) = b× log (Smn). Adjacency functions were obtained by approximate scale-free topology criterion. Then, we converted the adjacency matrix to the topology matrix. (2) Module detection: the modules with a minimum of 30 lncRNA/genes were identified using Dynamic Tree Cut method and Static Tree Cut method.

## Results

### Identification of DELs and DEMs

**Fig. 1:**
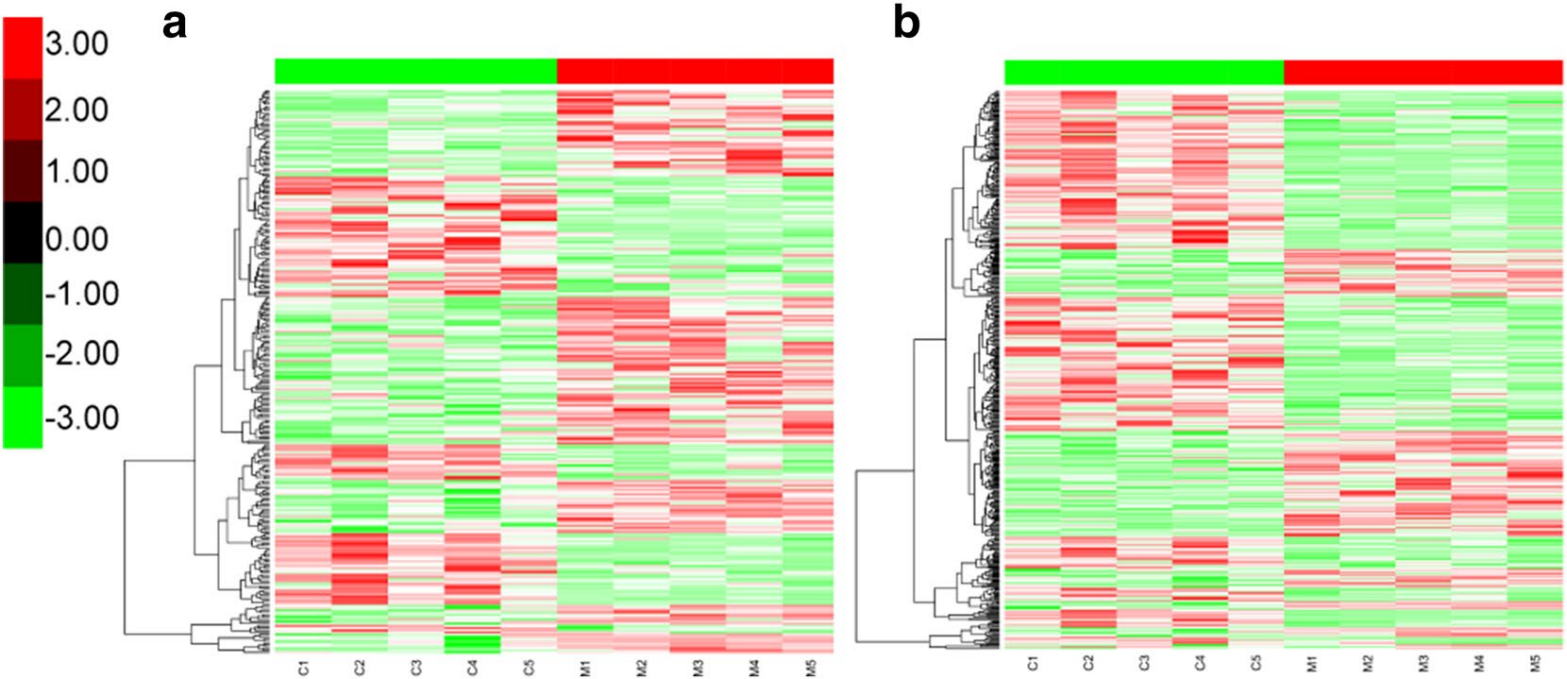
Hierarchical cluster analysis of differentially expressed lncRNAs and mRNAs. **a** Hierarchical cluster analysis of 254 differentially expressed lncRNAs. **b** Hierarchical cluster analysis of 472 differentially expressed mRNAs. Hierarchical cluster analysis showed that the differentially expressed genes ultimately clustered into two major branches, including up-regulated genes (labeled in red) and down-regulated genes (labeled in green)

According to our filtering criteria FC > 1.5 and *P* < 0.05, a total of 254 DELs, including 107 significantly up- and 147 down-regulated lncRNAs; 472 DEMs, including 308 significantly up- and 164 down-regulated mRNAs were screened. Hierarchical clustering was conducted to evaluate the altered lncRNAs and mRNAs expression pattern among samples (Fig. 1).

### GO and KEGG analyses

**Fig. 2:**
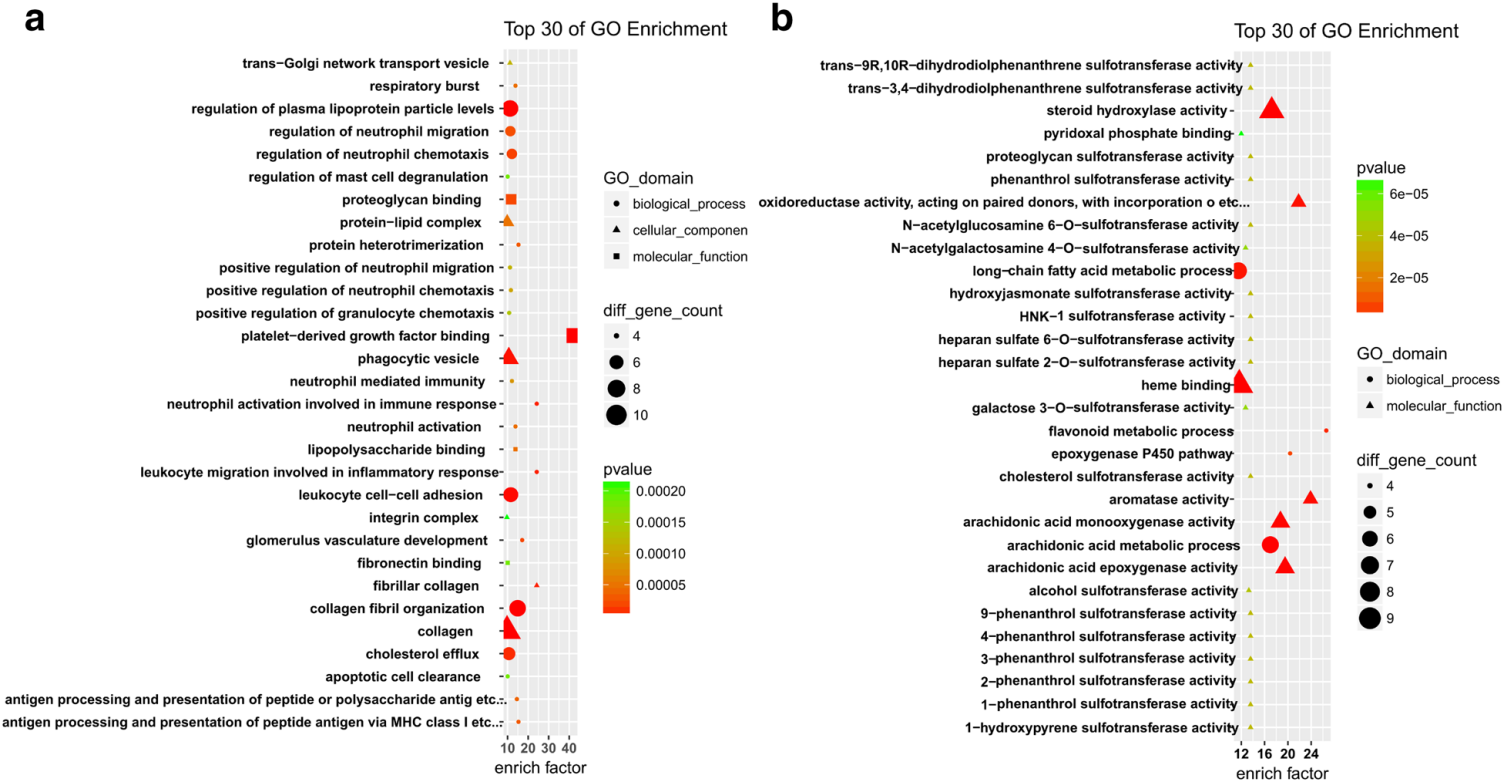
GO analysis of differentially expressed genes. **a** The top 30 GOs of the up- regulated genes. **b** The top 30 GOs of the down-regulated genes. The different colors from green to red represent the *P* value. The different sizes of the shapes represent the gene count

**Fig. 3:**
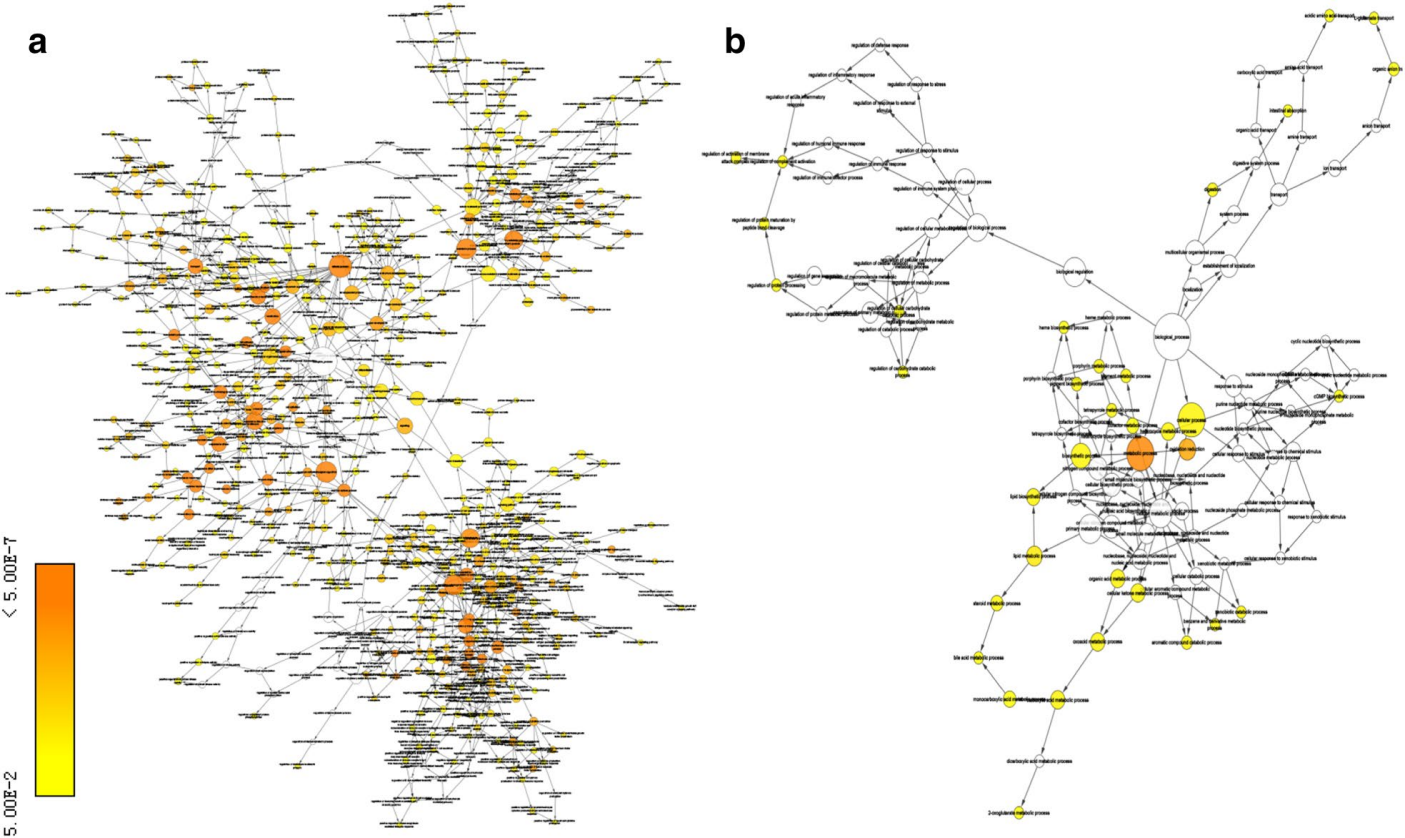
Go-net analysis of DE mRNAs. **a** Go-net of the up-regulated genes. **b** Go-net of the down-regulated genes. Yellow nodes: nodes with *P*-value < 0.05 and Benjamini corrected *P*-value < 0.05. The color of node is more deep, the functional difference is more significant

**Fig. 4:**
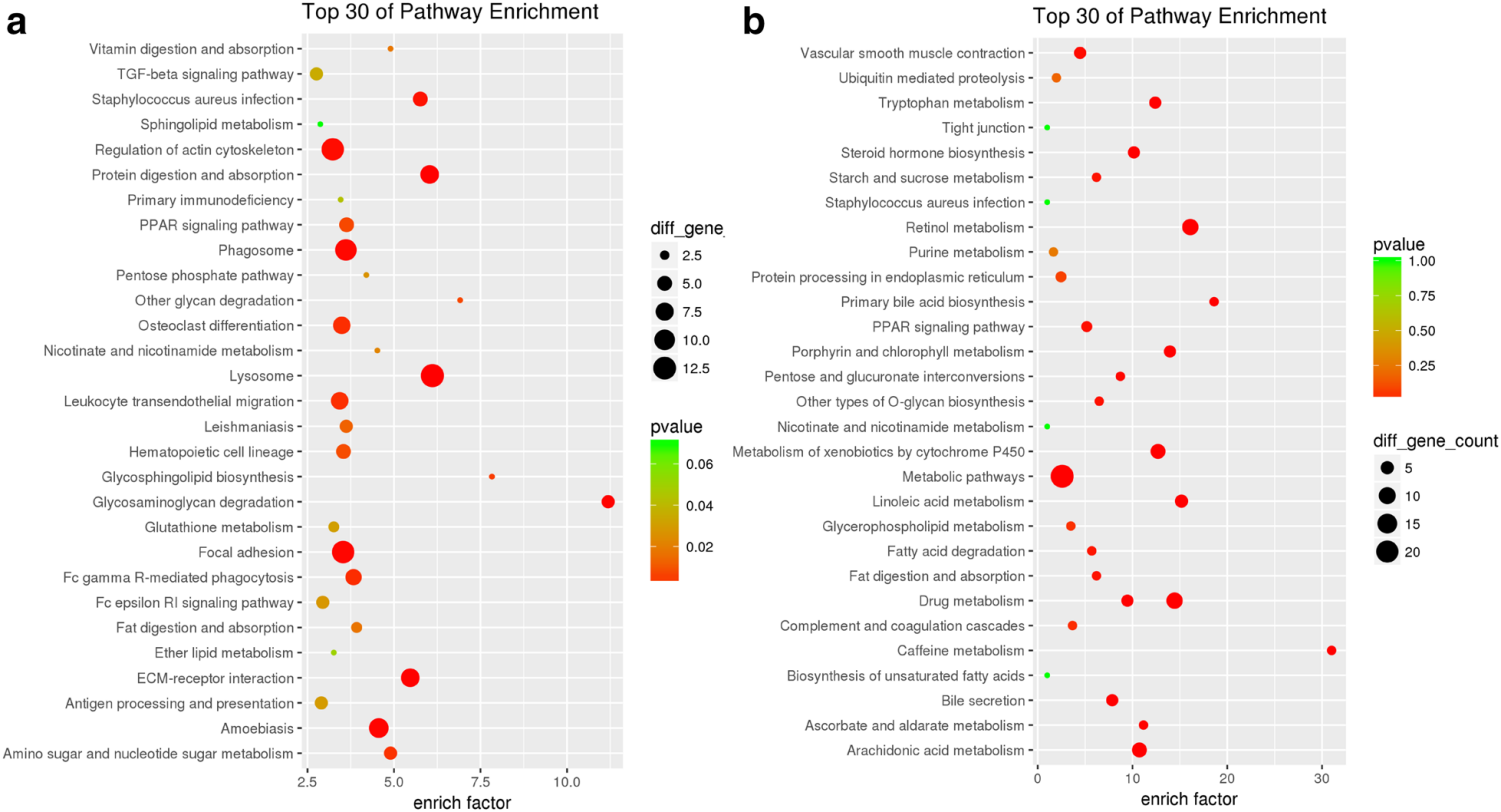
KEGG analysis of differentially expressed genes. **a** The top 30 pathways of the up-regulated genes. **b** The top 30 pathways of the down-regulated genes. The different colors from green to red represent the *P* value. The different sizes of the shapes represent the gene count

GO and KEGG pathway enrichment analyses were performed to determine the functions of the identified DEMs. GO analysis revealed that a total of 1117 GO terms were regulated by the up-regulated mRNAs, while the down-regulated mRNAs were enriched in 376 GO terms. The top 30 GO terms by the up- and down-regulated mRNAs were displayed in Fig. 2. The up GO-net and down GO-net were shown in Fig. 3. Pathway analysis demonstrated that 128 and 66 pathways were regulated by the up-regulated and down-regulated mRNAs, respectively. The top 30 significant pathways were shown in Fig. 4.

### PPI network

**Fig. 5:**
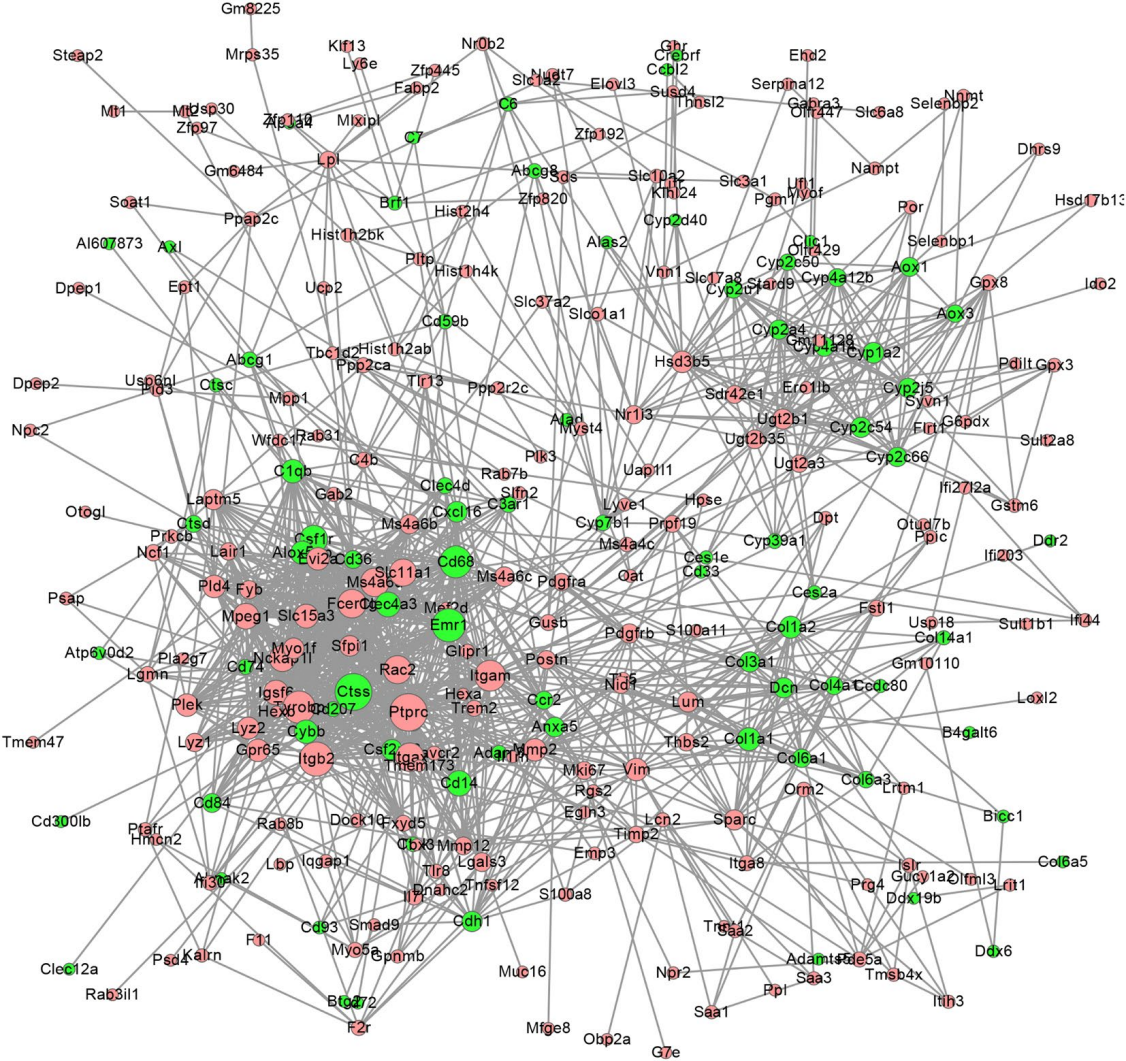
PPI network of DE genes constructed using Cytoscape software. The red nodes represent up-regulated genes, and the green nodes represent down-regulated genes. The edges represent the relationships between genes. The degree of one gene means the number of its interactions with other genes. The bigger one node indicates the higher connectivity degree. The higher connectivity degree stands for the more important role in the PPI network

After removing isolated genes, the PPI network of significantly up- and down- regulated genes was delineated using the STRING database. As shown in Fig. 5, the PPI network contained 284 nodes and 1269 edges. Genes with a high degree of connectivity in the PPI network may be potential targets for disease [20]. Genes in the top 10 for the highest degree of connectivity were displayed in Table 1, suggesting that these genes may play crucial roles in the origin and development of HF.

**Table 1:** Genes with top 10 highest connectivity degree in PPI

Gene	Node-degree	FC	*P*-value	Trend
Ptprc	56	1.84	0.0295	Up
Ctss	55	1.53	0.0005	Down
Itgb2	49	1.57	0.0151	Up
Adgre1	47	2.14	0.0169	Down
Tyrobp	45	3.13	0.0159	Up
Cd68	44	1.65	0.0404	Down
Itgam	42	1.57	0.0039	Up
Fcer1g	37	1.51	0.0403	Up
Itgax	37	1.57	0.0409	Up
Ms4a6d	37	1.64	0.0288	Up

### Construction of lncRNA-mRNA WGCNA

**Fig. 6:**
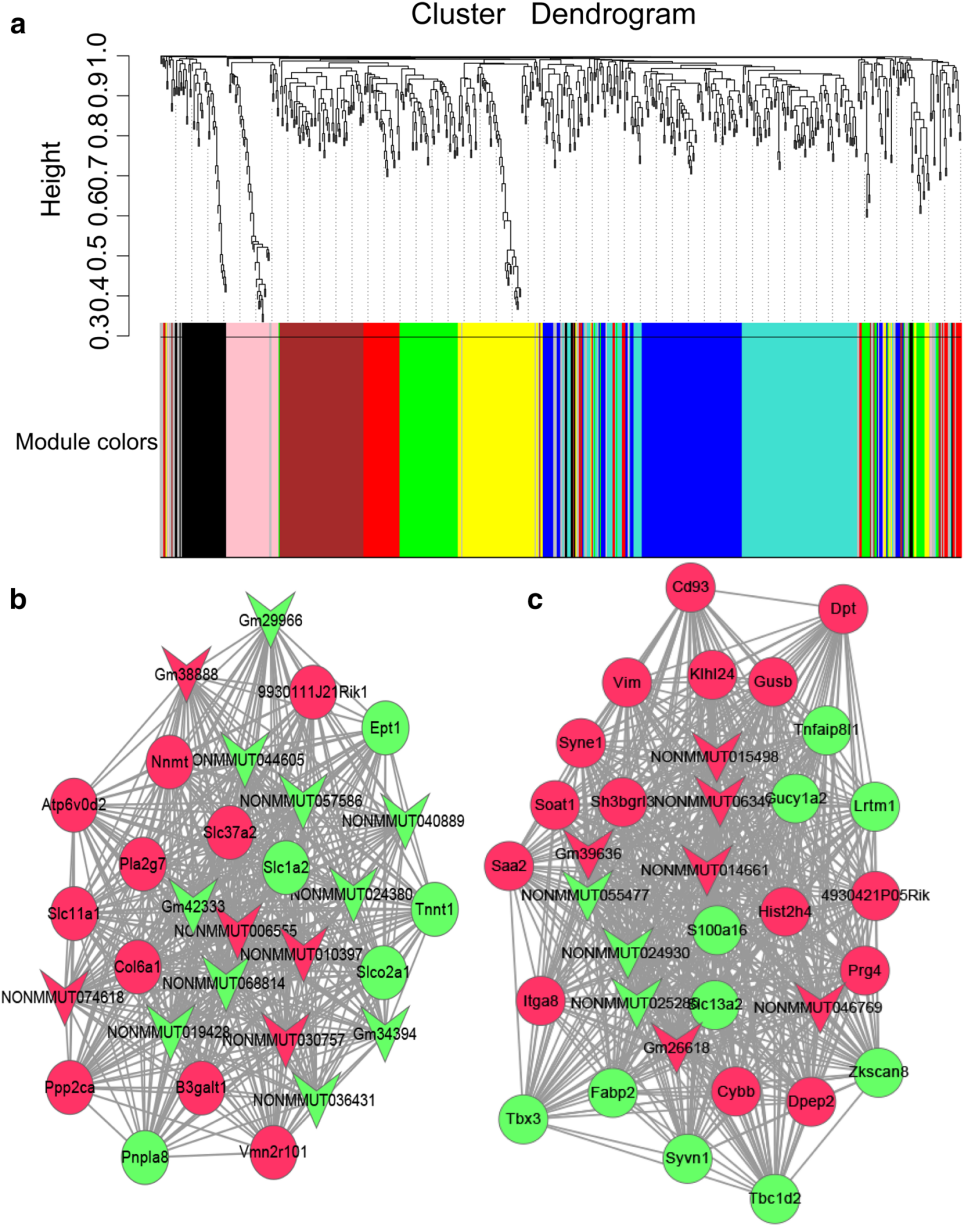
Cluster dendrogram and lncRNA-mRNA co-expression sub-networks. **a** Cluster dendrogram. Gene dendrogram showing the co-expression modules defined by the WGCNA labeled by colors. **b** Blue mode. **c** Turquoise module. Arrow nodes represented lncRNAs, roundness nodes represented mRNAs, red and green color represented up-regulated down-regulated, respectively

The lncRNA-mRNA co-expression network was established to investigate the association between DELs and DEMs. Via WGCNA package in R, we constructed a Cluster Dendrogram (Fig. 6a), then two weighted co-expression sub-networks were identified. The lncRNA-mRNA weighted co-expression network was constructed based on the genes with the top 30 connectivity degrees in two modules and *P*-value < 0.01. Among these, 24 lncRNAs and 40 mRNAs were involved in the two modules (Fig. 6b and c). The lncRNAs in the blue and turquoise modules were displayed in Tables 2 and 3, respectively.

**Table 2:** lncRNAs in blue module

lncRNA	Trend	*P* value	FC	Strand	Location
Gm38888	Up	0.01188	1.73	+	chr6
NONMMUT006555	Up	0.03700	3.22	–	chr10
NONMMUT074618	Up	0.00774	1.83	+	chrx
NONMMUT010397	Up	0.00094	1.72	+	chr11
NONMMUT030757	Up	0.01216	1.78	–	chr17
Gm29966	Down	0.01487	1.58	+	chr18
Gm42333	Down	0.03374	2.34	–	chr4
Gm34394	Down	0.02833	1.60	+	chr15
NONMMUT044605	Down	0.02778	1.55	+	chr3
NONMMUT057586	Down	0.01466	1.68	–	chr6
NONMMUT040889	Down	0.02319	1.72	–	chr2
NONMMUT024380	Down	0.04125	2.05	+	chr15
NONMMUT036431	Down	0.02754	1.56	–	chr2
NONMMUT068814	Down	0.02868	1.76	–	chr9
NONMMUT019428	Down	0.00494	2.29	+	chr13

**Table 3:** lncRNAs in turquoise module

lncRNA	Trend	*P* value	FC	Strand	Location
Gm39636	Up	0.00008	4.54	–	chr1
Gm26618	Up	0.01691	2.03	–	chrx
NONMMUT015498	Up	0.03700	3.22	–	chr10
NONMMUT06347	Up	0.00919	2.01	–	chr7
NONMMUT014661	Up	0.02384	1.57	+	chr12
NONMMUT046769	Up	0.01214	1.56	+	chr4
NONMMUT055477	Down	0.02489	1.58	–	chr5
NONMMUT024930	Down	0.03686	2.56	+	chr15
NONMMUT025285	Down	0.00742	1.99	–	chr16

### GO and KEGG analyses of the blue and turquoise modules

**Fig. 7:**
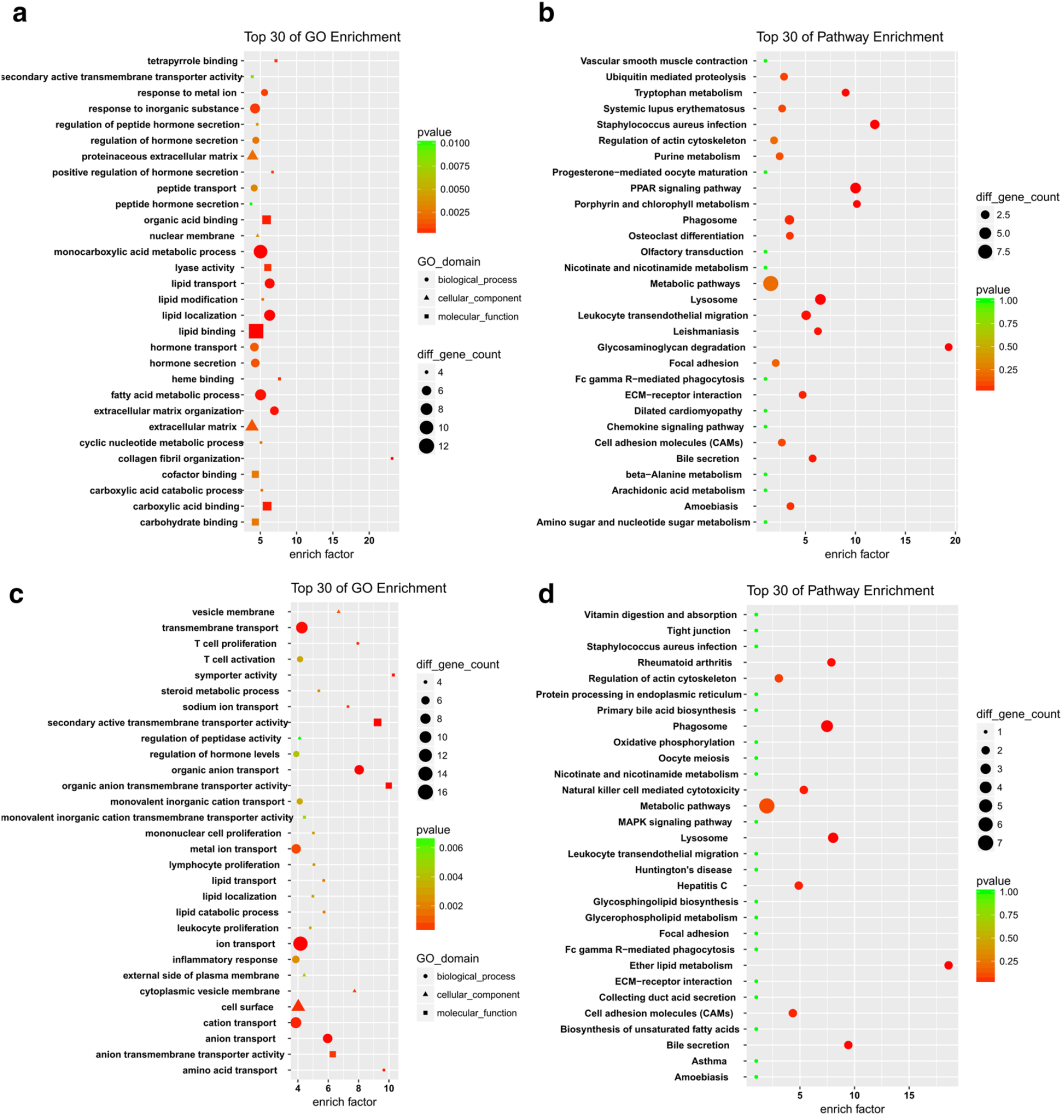
Functional annotation and pathway analysis for the differentially expressed mRNAs in the blue and turquoise modules. The top 30 GO annotations of the differentially expressed mRNAs in **a** the blue module and **c** the turquoise module are presented. The top 30 KEGG pathways from enrichment analysis in **b** the blue module and **d** the turquoise module are presented. The different colors from green to red represent the P value. The different sizes of the shapes represent the gene count

Furthermore, we performed GO and KEGG pathway annotations for the blue and turquoise modules. GO analysis revealed a total of 365 and 245 enriched terms in the blue and the turquoise modules, respectively. The top 30 terms are presented in Fig. 7a and c. KEGG analysis revealed that 67 and 48 pathways were enriched in the two modules, respectively. The top 30 pathways are presented in Fig. 7b and d.

## Discussion

Hepatic fibrosis, a reversible lesion, is a common progresses for acute or chronic liver diseases to liver cirrhosis, which is irreversible [21–24]. Therefore, it is crucial to explore the comprehensive mechanisms of HF to reverse its progress promptly. Over the past several years, as novel regulators in the cellular and biological processes, lncRNAs have attracted much close attention [25–27]. However, as previously mentioned, the number of meaningful key lncRNAs identified in HF tissues is still not sufficient.

In this study, we screened 254 DELs, which consisted of 107 up- and 147 down-regulated lncRNAs. Meanwhile, 472 DEMs were examined, which consisted of 308 up- and 164 down-regulated mRNAs. Furthermore, to investigate the functions of HF-related genes, we annotated these genes into GO and KEGG pathway analysis, we found that the DE genes were remarkably enriched in the GO terms involving TGF-β signaling pathway, ECM-receptor interaction, PPAR signaling pathway, etc. It is well known that activated hepatic stellate cells (HSCs) are famous for their role in liver fibrosis. Several studies have shown TGF-β plays a critical role in HSCs activation, and the TGF-β signaling pathway could be a potential therapeutic target for HF [28–30]. This classic signaling pathway is activated by TGF-β binding to its receptors located on the cell membrane. The downstream proteins, namely Smad2 and Smad3, are activated by phosphorylation, which further promotes the transcription of genes encoding ECM components, then accelerates the development of liver fibrosis [31]. Similarly, KEGG analysis revealed that the DE genes were mainly enriched in the metabolic process, which were closely related to previous studies. For example, our previous studies indicated that dysregulations of cytochrome P450, sphingolipid, glucose and water electrolyte, fatty acid, amino acid and energy metabolism might be involved in the pathogenesis of HF in rats [32, 33].

The WGCNA is a bioinformatics technology for describing the correlation patterns among genes across microarray samples. It is a powerful systems analysis technology that focuses on the coherent function of gene network modules, which aims at identifying higher-order relationships among gene products [34]. WGCNA analysis of lncRNAs and mRNAs has been successfully utilized to screen functionally enriched modules involved in complex diseases [35–37]. Via WGCNA, two lncRNA-mRNA weighted co-expression sub-networks were identified, which included 24 key lncRNAs and 40 mRNAs.

Although several LncRNAs that could affect hepatic fibrosis were already reported including lnc LFAR1 [15], p21 [38], MALAT1 [39], MEG3 [40] and so on. However, the functional characterization of the 24 key lncRNAs is still in its infancy. To infer potential function of LncRNAs, according to ceRNA theory, we studied the related to mRNAs, whose functions have been annotated. For example, lnc Gm38888, NONMMUT006555, NONMMUT074618 were screened out and could interact with Col6a1 in the blue module. It is worth noting that Col6a1, the main component of HF identified in our study, is a type VI collagen gene which has been widely reported to be closely associated with HF. Accumulation of type VI collagen may lead to the distorted architecture and functional damage to the liver in HF [41, 42]. Moreover, KEGG analysis showed that Col6a1 is enriched in ECM-receptor interaction, which has been reported to play a key role in HF, as discussed above.

For the turquoise module, lnc Gm39636, Gm26618, NONMMUT015498, etc., which interact with Itga8. In particular, Itga8 is a specific cell surface marker of mesothelial cell (MC)-derived HSCs and MCs. Itga8^+^ (MCs) is the maintain phenotype of hepatoblasts in liver development and myofibroblasts increase the expression of Itga8 during liver injury [43]. Recently research suggested that MCs played important roles in liver development, fibrosis, and regeneration [44]. KEGG analysis showed Itga8 is enriched in the pathways of ECM-receptor interaction, FA and PI3K-Akt signaling pathway, which have been demonstrated to be closely related with HF.

## Conclusion

In summary, HF was a complicated process with some lncRNAs, mRNAs involved, which were the key genes in contributing to pathogenesis of HF. This study revealed crucial information on the molecular mechanisms of HF and laid a foundation for subsequent gene validation and functional studies, which could contribute to the development of novel diagnostic markers and provide new therapeutic targets for the clinical treatment of HF. However, there were still some limitations in this study. Firstly, the key genes need to be verified in liver tissue specimens of liver fibrosis by RT-PCR. Secondly, further experiments are required to validate their effects and mechanisms in HF. The last but not least, the homology of the key genes needs to be validated for using as potential biomarkers and therapeutic targets in clinical applications.

## Data Availability

The datasets used and/or analyzed during the present study are available from the corresponding author on reasonable request.
